# Neonatal Piglets Are Protected from Clostridioides difficile Infection by Age-Dependent Increase in Intestinal Microbial Diversity

**DOI:** 10.1128/Spectrum.01243-21

**Published:** 2021-09-22

**Authors:** Alexandra Proctor, Nancy A. Cornick, Chong Wang, Shankumar Mooyottu, Paulo A. Arruda, Kayce Kobs, Gregory J. Phillips

**Affiliations:** a Department of Veterinary Microbiology and Preventative Medicine, Iowa State University, Ames, Iowa, USA; b Department of Production Animal Medicine, Iowa State University, Ames, Iowa, USA; c Department of Pathology, Iowa State University, Ames, Iowa, USA; University of Nevada Reno

**Keywords:** pig microbiota, *Clostridioides difficile* infection, colonization resistance

## Abstract

While Clostridioides difficile is recognized as an important human pathogen, it is also a significant cause of gastroenteritis and associated diarrhea in neonatal pigs. Since clinical disease is rarely diagnosed in piglets older than 1 week of age, it is hypothesized that natural resistance is associated with the increased complexity of the intestinal microbiota as the animals age. To test this, piglets were challenged with C. difficile (ribotype 078/toxinotype V) at times ranging from 2 to 14 days of age, and the severity of disease and microbial diversity of the cecal microbiota were assessed. Half of the piglets that were challenged with C. difficile at 2 and 4 days of age developed clinical signs of disease. The incidence of disease decreased rapidly as the piglets aged, to a point where none of the animals challenged after 10 days of age showed clinical signs. The cecal microbial community compositions of the piglets also clustered by age, with those of animals 2 to 4 days old showing closer relationships to one another than to those of older piglets (8 to 14 days). This clustering occurred across litters from 4 different sows, providing further evidence that the resistance to C. difficile disease in piglets greater than 1 week old is directly related to the diversity and complexity of the intestinal microbiota.

**IMPORTANCE** C. difficile is an important bacterial pathogen that is the most common cause of infections associated with health care in the United States. It also causes significant morbidity and mortality in neonatal pigs, and currently there are no preventative treatments available to livestock producers. This study determined the age-related susceptibility of piglets to C. difficile over the first 2 weeks of life, along with documenting the natural age-related changes that occurred in the intestinal microbiota over the same time period in a controlled environment. We observed that the populations of intestinal bacteria within individual animals of the same age, regardless of litter, showed the highest degree of similarity. Identifying bacterial species associated with the acquisition of natural resistance observed in older pigs could lead to the development of new strategies to prevent and or treat disease caused by C. difficile infection.

## INTRODUCTION

Clostridioides difficile is a Gram-positive, spore-forming bacterium that is a significant cause of enteric disease in humans, as well as a wide variety of mammals (1–3). C. difficile is ubiquitous in the environment and frequently found as a member of the mammalian gastrointestinal (GI) microbiota (4, 5). Of particular importance to food animal agriculture, C. difficile infection (CDI) can lead to potentially fatal gastroenteritis in neonatal piglets. Strains belonging to ribotype 078/toxinotype V account for approximately 90% of the isolates recovered from piglets with significant disease confined to the neonatal period, generally 2 to 5 days after birth (6–9). Since C. difficile can be cultured from both healthy animals and those with diarrhea, diagnosis of CDI requires demonstration of toxins TcdA and/or TcdB, as well as observation of macroscopic and histopathologic intestinal lesions in the spiral colon (3, 10). The majority of neonate piglets are culture positive for C. difficile, but the intestinal population of the pathogen appears to decline over the first 2 months of life (8, 11). Mechanistically, it is unknown why C. difficile-associated disease is confined to neonate piglets. One hypothesis is that host resistance to CDI results directly from an increasing diversity of the GI microbiota, which may provide protection by competitive exclusion through a reduction in available niches, nutrient availability, or production of antimicrobial metabolites (12).

The mammalian GI tract is colonized by a complex community of bacterial taxa that imparts several benefits to the host, including nutrient acquisition, colonization resistance, and immunomodulation (13). The microbial community also changes in response to diet, age, and host health status. Age-related changes in the microbiota occur over the lifetime of the host and are more prominent in younger animals soon after birth and again at weaning (14, 15). A succession of increasing levels of microbial diversity of the intestinal microbiota begins as neonatal animals are exposed to the maternal microbiota and to microbes present in their environment (12, 16). Intestinal microbial diversity and complexity continues to change from the time of weaning as the diets of young animals shift to solid feed that includes complex carbohydrates (14).

Previous studies to profile the microbial diversity in pigs have included characterization of piglets at weaning (3 weeks of age) and older (12, 17–19), but fewer studies have investigated C. difficile disease and the microbiota longitudinally in young, nursing piglets (15, 20, 21). Since colonization of pigs by C. difficile declines within a relatively short window of time as the animals age (8), it is important to identify host factors that contribute to colonization resistance against the pathogen. To better understand how the host gut microbiota is associated with C. difficile colonization and disease, we used 16S rRNA gene amplicon sequencing to track the changes in the taxonomic composition of the GI microbiota in neonatal piglets during the first 14 days of life in a controlled environment. We further associated the changes in the microbiota composition with the emergence of natural resistance to CDI as the pigs aged.

## RESULTS

Table 1 summarizes the design of the two separate experiments conducted in this study, as described in Materials and Methods. In each experiment, neonatal piglets were challenged with C. difficile (ribotype O78) spores at 2, 4, 6, 8, 10, 12, or 14 days of age. At 2 days postinfection, challenged piglets and one nonchallenged control piglet of the same age were euthanized. Cecal contents were collected for 16S rRNA gene amplicon sequencing, and contents from the spiral colon were collected for TcdA/TcdB toxin detection. Additionally, tissue from the cecum and spiral colon was taken for histological examination.

**TABLE 1:** Experimental design of study

Expt	Sows (2 per expt)	No. of:		Inoculum dose	No. of control animals	Nutrient source for control animals
		Piglets	Challenged piglets			
1	1 and 2	24	19	10^3^	5	Nursing
2	3 and 4	28	10	5 × 10^4^	3	Milk replacer
			15	10^6^		

We used the presence of edema and microscopic lesions of mesocolonic tissue as indicators of disease, along with the presence of toxin, since lesions do not occur in the absence of toxin during the course of the disease. The results of this analysis are shown in Table 2. At necropsy, signs of disease and/or toxin were observed exclusively in piglets challenged at the earliest ages, which was consistent with previous studies (22). Lesions can be segmental, and since we did dissect multiple sections of tissue, it is likely that additional lesions were missed by chance alone. Regardless, all of the piglets ≤6 days of age that were toxin positive were symptomatic. Specifically, half of the piglets (6/12) that received the spore challenge at either 2 or 4 days of age displayed evidence of disease (Table 2), including classic histopathologic lesions and the presence of toxins A and B within cecal contents (10, 23). In contrast, only a minority of piglets challenged at 6 or 8 days of age showed evidence of disease (3/16), and none of the piglets challenged at ≥10 days of age displayed clinical signs associated with CDI (0/14). None of the unchallenged control piglets was symptomatic, and they had unremarkable histologic examination. Furthermore, no toxins were detected in the cecal contents (Table 2).

**TABLE 2:** Observations of toxin and gross and microscopic lesions in piglets challenged with C. difficile at 2 to 14 days old

Age (days)	No. of piglets with symptom/total no. of piglets			% symptomatic
	Mesocolonic edema	Toxin positive	Microscopic lesions	
2	3/6	3/6	1/6	50
4	0/6	3/6	0/6	50
6	1/8	2/8	1/8	25
8	0/8	1/8	0/8	12.5
10	0/8	0/8	0/8	0
12	0/3	0/3	0/3	0
14	0/3	0/3	0/3	0
Uninfected controls	0/8	0/8	0/8	0

Culturing directly from cecal contents yielded toxigenic C. difficile from four challenged piglets, all inoculated at 6 days or younger. In contrast, C. difficile was not recovered from the older piglets or control piglets. Because CDI rarely occurs in piglets older than 1 week, we grouped piglets into younger (days 2 to 8) and older (days 10 to 14) ages for statistical analysis of the presence of CDI. Fisher’s exact test yielded a *P* value of 0.0186.

We next assessed the abundances of individual bacterial taxa from the cecal contents by 16S rRNA gene amplicon sequencing. For this, we analyzed microbial abundance data from piglets grouped by the age at which they were inoculated to identify any changes in the composition of the gut microbiota over time. While we initially compared the results from experiment 1 to those of experiment 2 (Table 1), we found no significant differences in abundance; therefore, the data were combined to increase statistical rigor for further analysis. Although diet is known to impact the gut microbiota, we failed to observe significant differences between the samples recovered from the control animals, regardless of diet (Table 1); therefore, the data from these animals were also combined. Since all of the piglets received colostrum before being challenged or removed from the sow, there was apparently insufficient time for changes between the two groups of control animals to be detected using 16S rRNA gene amplicon sequencing (Table 1).

**FIG 1:**
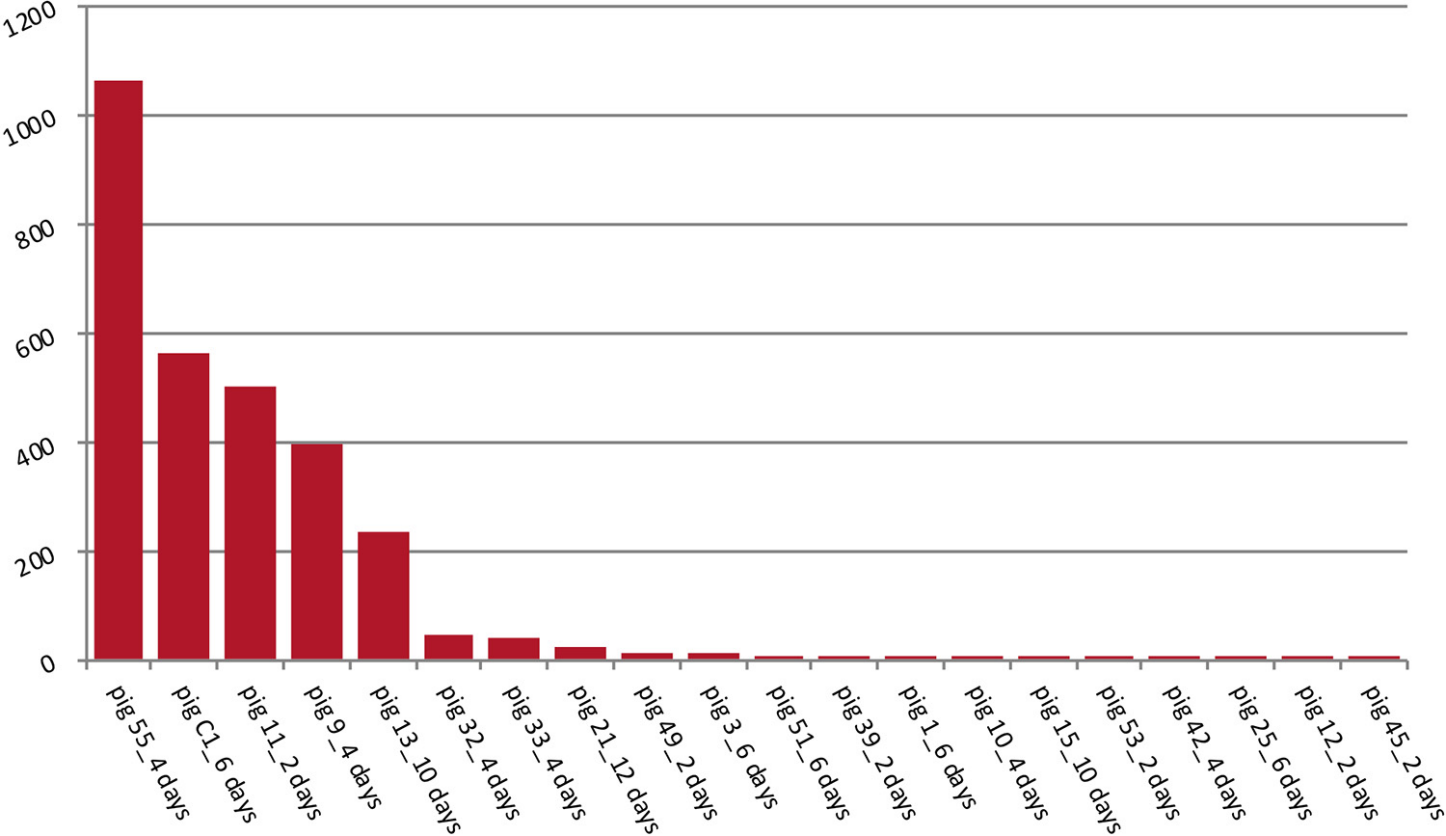
Read counts that match the C. difficile 16S rRNA gene sequence. Sequences matching C. difficile were found primarily in young piglets. While a few control animals also had C. difficile sequences, these animals showed no signs of disease and likely acquired C. difficile from the sows.

Collectively, we found no significant differences in bacterial diversity or taxon abundance between piglets that were challenged with C. difficile spores and the negative controls (data not shown). While the numbers of sequences that matched the V3-V4 variable region of the C. difficile 16S rRNA gene were highly variable (Fig. 1), the results revealed the presence of the pathogen in approximately half of the piglets and primarily in younger animals. C. difficile sequence signatures were observed in 4/5 piglets challenged at 2 days of age, 6/6 challenged at 4 days of age, 6/8 challenged at 6 days, and 5/24 challenged at a later age. C. difficile sequence signatures were also observed in 3/8 unchallenged control animals. Since the control piglets showed no signs of disease, it is likely that the animals acquired C. difficile from the sow during parturition, since approximately 40% of sows shed C. difficile (8, 22, 24, 25).

### Relative abundances of cecal bacteria.

**FIG 2:**
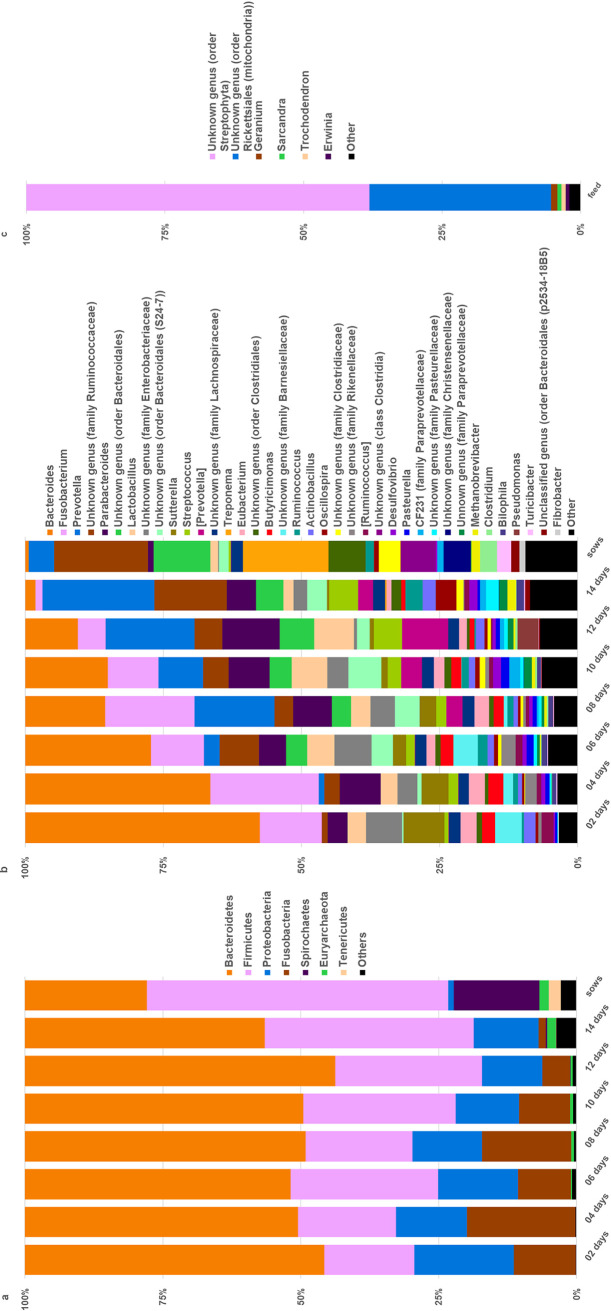
Relative abundance measurements for selected phylogenic levels in each age group. The relative proportions of individual taxa are shown for samples from animals ranging from 2 to 14 days old. The taxonomic compositions of the microbiota from the feed and sows are also shown for comparison. OTUs making up less than 1% in each sample were binned as “Other.” Taxa are shown by the key on the right for phylum (a), for genus (b), and for feed at the genus level (c). At the phylum level (a), *Bacteroidetes* was the most abundant phylum in the piglets, followed by *Firmicutes*. *Firmicutes* was the most abundant phylum in the sows, followed by *Bacteroidetes*. At the genus level (b), the numbers of taxa appeared to increase as the piglets aged, to more closely resemble the sows. C. difficile was not present in the feed sample (c), indicating that the only exposure the piglets received was through experimental infection.

A total of 1,868,925 reads from 57 samples, with an average of 32,788 ± 7,545 (mean ± SD) sequences per sample, generated 4,438 total observations, with a minimum number of reads of 16,822 and a maximum of 48,706. Figure 2 summarizes the taxonomic abundances for piglets at each age postinoculation at the phylum (Fig. 2a) and genus (Fig. 2b) levels, as well as for the feed provided to the sows (Fig. 2c). As shown, the microbiota of the piglets was dominated by *Bacteroidetes* (43.5 to 56.2%) and *Firmicutes* (16.3 to 37.85%), which is consistent with previous reports (26, 27). Also, *Bacteroidetes* decreased while *Firmicutes* increased as the pigs aged. While these shifts were not considered significant for the short duration of this experiment, they suggest a trend in which the microbiota compositions of the piglets would have become more similar to those of the sows (22.1% for *Bacteroidetes* and 54.7% for *Firmicutes*) had the animals been allowed to mature (Fig. 2a). Within the phylum *Bacteroidetes*, *Bacteroidia* (43.5 to 55.9%) was the major class observed in the piglets, but at a reduced level by 14 days of age. Members of the *Bacilli* (4.3 to 12.6%), *Erysipelotrichia* (2.0 to 3.1%), and *Clostridia* (9.6 to 28.6%) were the dominant classes in the phylum *Firmicutes*. *Bacteroidia* and *Bacilli* also decreased as the piglets aged, along with an increase in the *Clostridia* (data not shown). The most notable differences occurred between the piglets and sows or between very young piglets and older piglets. This trend was further observed at the genus level (Fig. 2b), as *Bacteroides* decreased and genera in the phylum *Firmicutes* increased in the older piglets to a point where the 14-day-old piglets appeared more similar to the sows than did the 2-day-old piglets.

### Alpha and beta diversity.

**FIG 3:**
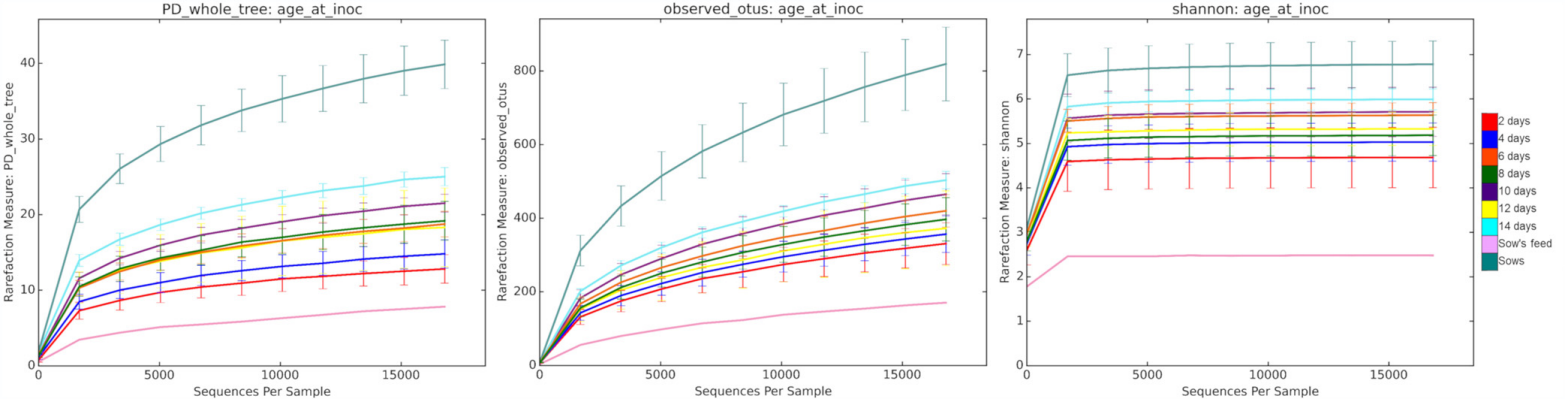
Mean bacterial diversity (alpha diversity) of the cecal microbiota of piglets by age. Each line represents all of the piglets challenged at a specific day of age, as indicated by the key on the right. Left, Faith’s phylogenetic diversity (PD). A general increase in diversity, as measured by branch lengths, was observed as the piglets aged. Middle, the observed operational taxonomic unit (OTU) metric increased as piglets aged, indicating an increase in the diversity of bacteria present as the piglets matured. Right, Shannon indices also showed an increase as the piglets aged. Error bars show standard deviations.

Alpha diversity (Fig. 3) was calculated using QIIME and compared using the two-sample *t* test with *P* values calculated using Monte Carlo permutations and Bonferroni correction for multiple comparisons. Faith’s phylogenetic diversity (Fig. 3a) revealed increasing microbial diversity as the piglets aged, with significant differences between days 2 and 6, 2 and 8, and 4 and 10 (*P* = 0.036, for each increment). Although the microbial diversity in piglets at 2 and 4 days of age appeared to be distinct from that of the sows, the differences were not considered significant (*P* = 0.072, for both). However, piglets at ages 6, 8, 10, and 12 days were significantly different from the sows (*P* = 0.036, for each increment). The observed-OTU metric (Fig. 3b) followed a similar pattern, with increasing numbers of observed OTUs in the older piglets compared to the numbers in younger piglets, with day 2 being significantly different from days 6 and 10 (*P* = 0.036, for both) and piglets at ages 2, 4, 6, 8, and 10 days being significantly different from the sows (*P* = 0.036, for all). Shannon diversity (Fig. 3c) also showed an increase as piglets aged, with piglets at ages 2 and 4 days being significantly different from the sow (*P* = 0.036, for both).

**FIG 4:**
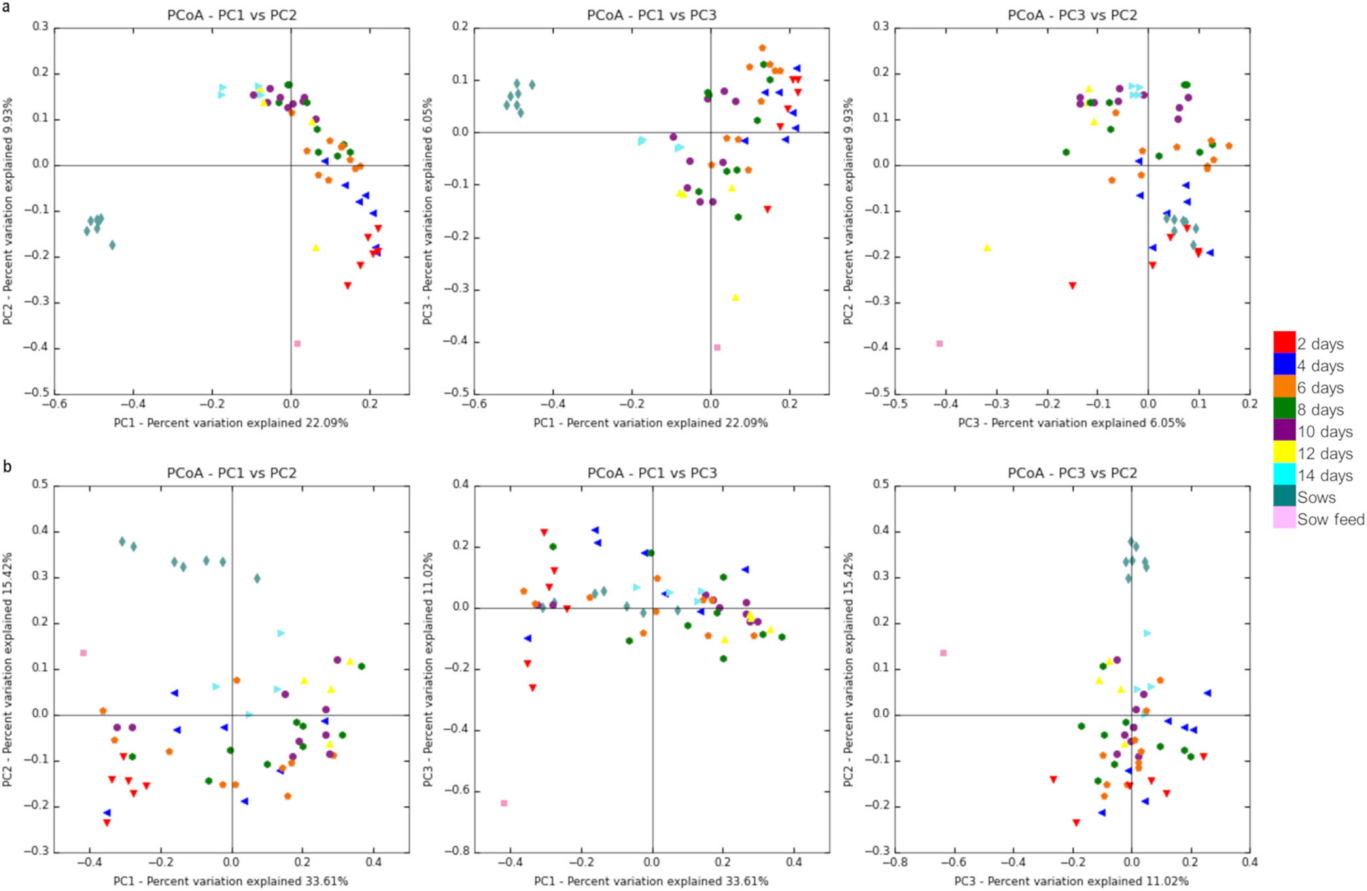
Relative differences (beta diversity) between the cecal microbiota of individual piglets. Each point in the principle coordinate analysis (PCoA) graphs represents an individual animal, and each color represents the age of the animal at the time of challenge with C. difficile, as specified by the key on the right. (a) Unweighted UniFrac analysis. Clustering of the piglets by age is observed, with a taxonomic progression that moved toward the microbiota composition of the sows. The same pattern of clustering was observed over 4 litters of piglets. (b) Weighted UniFrac analysis. Similar to the unweighted UniFrac analysis, clustering of animals of similar age was observed, albeit with greater variability.

Unweighted UniFrac analysis indicated that the microbial community from the cecum of individual piglets clustered by age and shifted toward that of the sows (Fig. 4a), with an analysis of similarity (ANOSIM) test statistic of 0.65 and *P* = 0.001. The microbial communities of piglets challenged at 2 and 4 days of age were more similar to one another than to those of older piglets. Piglets that were 6 days of age at challenge clustered with 8-day-old piglets. Older piglets challenged at 10, 12, and 14 days of age clustered together. This clustering occurred across 4 different litters of piglets.

UniFrac analysis (Fig. 4) was conducted to reveal patterns of similarity in microbial composition with the age of the piglets. Both unweighted (Fig. 4a) and weighted (Fig. 4b) UniFrac analyses showed similar clustering, with the latter showing a less distinct clustering than unweighted (ANOSIM statistic of 0.41 and *P* = 0.001). Both metrics showed that piglets challenged at 2 and 4 days of age clustered more closely together than they did to the older piglets. The older piglets challenged at 6, 8, 10, 12, and 14 days of age also clustered more closely together. The more distinct clustering of same-aged piglets in the unweighted UniFrac compared to the weighted UniFrac suggests greater variability in the abundance of the microbiota between piglets of the same age. It is interesting to note that the one 8-day-old piglet that showed disease clustered with the 2-day-old piglets.

Pairwise ANOSIM statistical tests were conducted between age groups to determine which ages were different from each other (Table S1 in the supplemental material). Piglets of similar ages were more alike than the more distant younger or older pigs. For example, comparing piglets at 2 and 4 days of age, the unweighted ANOSIM test statistic was 0.18 with a *P* value of 0.032 and the weighted test statistic was 0.29 with a *P* value of 0.028. Comparing piglets aged 2 versus 14 days of age, however, yielded an unweighted ANOSIM test statistic of 0.92 with a *P* value of 0.004, with a weighted test statistic of 0.97 and a *P* value of 0.008. All of the age groups were significantly different from the sows.

### Predicted microbiome functional analysis with PICRUSt.

The predicted functional capacity of the microbiota of the piglets was determined using PICRUSt, and functional differences among age groups were tested using statistical analysis of metagenomic profiles (STAMP) (28, 29). This analysis yielded several categories of microbiome/host functions that were predicted to differ significantly between the experimental animal groups based on the composition of the microbiota (Table S1). Specifically, broad categories of carbohydrate, nitrogen, energy and amino acid metabolism, secondary metabolite biosynthesis, and cell signaling pathways were found to differ between animals grouped as young or old. Differences in more specialized functional categories were also noted, including functions associated with bacterial toxins and glycosaminoglycan degradation, which showed elevated activity in the younger piglets (*P* = 0.017, *P* = 0.01 and 0.012, respectively). Likewise, signatures of sphingolipid metabolism (*P* = 0.008) and steroid hormone biosynthesis (*P* = 0.007) were also higher in younger piglets than in older piglets and decreased with increasing age.

## DISCUSSION

To better replicate current animal production standards, we designed the experiments to maintain piglets with the sows for at least 48 h until beginning C. difficile challenges. At that time, some of the controls were fed milk replacer, while others were left to nurse from the sow until necropsy (Table 1). However, no significant differences in taxonomic abundance were observed over the short time course of this study. For the symptomatic piglets, we defined CDI as the presence of lesions or the presence of toxin. While it is well documented in human neonates that toxin can be present in asymptomatic individuals (3, 30), we are not aware of any studies that demonstrate the presence of toxin in asymptomatic piglets. Given this, we include the presence of toxin in symptomatic neonatal piglets as an indicator of CDI, since other major causes of diarrhea had been eliminated through vaccination of the sows.

Under these experimental conditions, half of the piglets (6/12) challenged at 2 or 4 days of age showed evidence of disease. The disease waned, however, by 6 and 8 days of age, as only 3/16 animals showed clinical signs. Evidence of disease became nonexistent by 10 days of age. While CDI has been observed in 10-day-old piglets, it requires specific experimental conditions where the piglets are removed from the sow within a few hours of birth and fed solely milk replacer, with minimal colostrum (20, 22). These results suggest a role of the developing gut microbiota in establishing colonization resistance to C. difficile in pigs and are consistent with other published studies showing that clinical disease due to CDI is highly age dependent and limited to neonate piglets (7, 8, 11).

Characterization of the gut microbiota over time in the growing piglets revealed that *Bacteroides*, *Fusobacterium*, *Enterobacteriaceae*, and *Sutterella* were the dominant microorganisms in younger animals and their abundances decreased with age. Around 1 week of age, *Prevotella* increased to become the dominant organism in the older piglets. This is in agreement with other studies where *Bacteroides* was more abundant in very young piglets and *Prevotella* was the dominant organism in the cecum and distal GI in piglets 7 days and older (19, 31). Specifically, while 16S rRNA gene sequence signatures matching *Prevotella* made up less than five percent of the relative abundances in animals at day 6, they increased to 20% in the older pigs. *Prevotella* has also been shown to associate negatively with the abundance of C. difficile in young piglets (21) and could represent a taxon that contributes directly to the resistance of older piglets to CDI.

In contrast, *Fusobacterium* is a microorganism associated with disease and inflammation (32). Piglets with neonatal porcine diarrhea had twice as much *Fusobacterium* as their healthy counterparts (33). Yang, et al. also found *Sutterella* to be more abundant in neonatal piglets with diarrhea (34). Here, we saw that both *Fusobacterium* and *Sutterella* were more abundant in younger piglets and decreased with age and decreased susceptibility to CDI.

We also note parallels between the prevalence of CDI in humans and the pigs studied here. For example, it has been shown that several bacteria in the orders *Clostridiales* and *Erysipelotrichales* in non-CDI patients appeared to confer resistance to C. difficile compared to infected patients (35). In the study reported here, several genera from the order *Clostridiales* increased in abundance around 6 to 8 days of age, including unknown genera in the family *Ruminococcaceae*, as well as the genera *Ruminococcus* and *Oscillospira*. While the abundances of these taxa at 6 or 8 days of age were not significantly different from earlier time points, they suggest a trend toward increasing levels of the bacteria as the animals age. The absence or decreased abundance of *Prevotella* was also associated with CDI in humans (36).

While the roles of specific host and environmental factors affecting colonization resistance are not completely understood, the host microbiota clearly plays a significant role (37). In general, increased microbial diversity correlates with pathogen colonization resistance (37), including against C. difficile in pigs (21). This trend is also observed in humans and in animal models, as antibiotic treatment is typically required to establish C. difficile colonization in older and experimental animals with a more diverse microbiota (38, 39) and CDI is associated with antibiotic therapies in humans (40). Antibiotics can deplete specific taxa of the microbiota and decrease the overall diversity of the microbial community, and their use is associated with histological changes in the GI tract (41, 42).

The role of the microbiota in colonization resistance is multifactorial, and possible explanations include the possibility that a decrease in microbial abundance and complexity increases the availability of nutritional or spatial niches. Correspondingly, a decrease in complexity can reduce the levels of antimicrobials produced by members of the microbiota that may otherwise inhibit the germination or growth of C. difficile (43). CDI in young piglets could also be influenced by anatomical and epithelial host factors associated with early development that in turn could also affect the composition of the GI microbiota. Consistent with these explanations, we observed a significant change in alpha diversity as the piglets grew from 2 to 10 days (Fig. 4).

To further take advantage of the taxonomic abundance data, we conducted PICRUSt analysis to identify functional features of the microbiota that potentially contribute to disease resistance (Table S2). We highlight a few observations from this analysis that could help explain how differences in microbiota composition may influence disease. For example, we observed that younger piglets had a higher relative percentage of microorganisms predicted to degrade glycosaminoglycan than did older piglets, and the younger piglets experienced C. difficile-associated enterocolitis while the older piglets did not. The ability to degrade glycosaminoglycans is associated with colitis and may be a contributor to disease severity (44). Similarly, steroid hormone biosynthesis capability was predicted to be decreased in older animals. These hormones, including glucocorticoids, are associated with physiological stress, such as the immunological stress of colitis (45, 46). Sphingolipids are part of the host cell plasma membranes; their metabolism has been associated with intestinal inflammation and polyps in humans, and higher predicted levels of sphingolipids correlate with increased host epithelial cell damage in the younger piglets (47). Finally, we note that the predicted prevalence of the coding capability for bacterial toxins was higher in younger pigs, which appears to reflect the lower diversity with a higher proportion of potentially pathogenic *Enterobacterales* (data not shown).

The results presented here can also be considered in the light of improved strategies to bolster colonization resistance to C. difficile in young pigs. Because the incidence of CDI in piglets decreases with age, it stands to reason that manipulation of the piglets’ GI microbiota to increase diversity and promote GI morphological changes to resemble those of more mature animals could potentially reduce C. difficile colonization. Towards this, pre- and probiotics have been tested in postweaned animals. A study using both a *Lactobacillus* strain and swine-specific *Pediococcus* showed improved average daily gain in both growing and finishing phases, as well as increased crypt depth and villus height in the jejunum of the probiotic-treated animals compared to the crypt depth and villus height in controls (48). Lactobacillus fermentum, a swine-specific strain, was also shown to be protective against Escherichia coli infection, apparently by modulation of the immune system in newly weaned piglets, and also increased weight and feed conversion of the piglets (49). In addition, nontoxigenic strains of C. difficile administered to young piglets prior to oral challenge with virulent C. difficile resulted in lower prevalence of CDI in a controlled, experimental setting (50, 51). While preliminary studies using selected probiotics show promise in protecting preweaned piglets from disease, more studies are needed to assess the roles of other members of the gut microbiota in colonization and disease resistance. This can also include identifying autochthonous microorganisms present around 8 to 10 days of age that could prove useful in young animals to aid in prevention of C. difficile colonization through competitive exclusion. In addition, rearing strategies have been shown to influence microbiota diversity, with increases in diversity observed in animals who were reared in isolation and fed milk replacer (19). Interestingly, dietary exposure to soil to mimic the outdoor environment also accelerated the acquisition of microbial diversity, including *Prevotella* (26), which correlated positively with increased disease resistance in the study reported here (Fig. 2b).

We note too that C. difficile has been isolated from healthy animals of different ages and stages of production (8), and DNA sequences matching C. difficile were recovered from several control animals in the current study. These control animals were toxin negative, showed no mesocolonic edema, and did not have histologic lesions or diarrhea, suggesting the piglets likely acquired C. difficile from the sows.

In conclusion, the microbial diversity of the cecal contents increased with the age of the piglets. The clustering of the piglets by age in the UniFrac analyses is consistent with the hypothesis that resistance to C. difficile disease in animals greater than 1 week of age can be explained by the increased diversity and complexity of the intestinal microbiota. Despite the high animal welfare and economic impact associated with CDI in neonate piglets, no commercial vaccine or approved antibiotic treatments are commercially available. The identification of bacterial species or groups of bacteria associated with the development of natural resistance in older pigs could be the key to the development of new alternatives to prevent and/or treat disease.

## MATERIALS AND METHODS

### Animals.

Four pregnant sows vaccinated against E. coli and rotavirus (Merck Prosystems RCE) were obtained from a commercial source and farrowed in biosafety level 2 (BSL-2) large animal facilities. All animal experiments were conducted in accordance with policies of the Iowa State University Institutional Animal Care and Use Committee (IACUC). Two separate experiments were conducted, as summarized in Table 1. After birth, piglets were allowed to nurse *ad libitum* until challenged. At 2, 4, 6, 8, 10, 12, or 14 days of age, two piglets were randomly selected and inoculated with 10^3^ to 10^6^ heat-activated spores of C. difficile strain ISU 15454-1 using an intragastric tube. This strain belongs to ribotype O78, toxinotype V, and produces both TcdA and TcdB (22). Once challenged, piglets were housed in clean 18-gallon plastic tubs and fed milk replacer (Esbilac, 10 ml 3 times/day) for the remainder of the experiment. Uninoculated control animals either continued to be nursed by the sow (experiment 1) or were removed from the sow and fed milk replacer (experiment 2). At 72 h postinoculation, each pair of C. difficile-challenged piglets plus one unchallenged control piglet was euthanized and necropsied.

### Necropsy.

At necropsy, gross pathological changes were recorded and contents from the cecum were collected for bacterial culturing. Contents from the spiral colon were also collected for toxin analysis by enzyme-linked immunosorbent assay (ELISA) (experiment 1 and 2) as described previously (10) or for Vero cell assay (experiment 2) (10, 23). Cecal contents for 16S rRNA gene amplicon sequencing were frozen at −80°C until processing. In addition, sections of cecum and spiral colon were fixed in 10% formalin, processed for standard histological evaluation using hematoxylin and eosin staining, and scored by a veterinary pathologist (P.A.A.) blinded to the experimental design.

### DNA isolation and library preparation.

Total genomic DNA from piglet cecal contents, sow feces, and the sows’ feed was extracted using the MoBio PowerSoil DNA isolation kit (MoBio Laboratories, Carlsbad, CA, USA). PCR amplification of the V4 variable region of the 16S rRNA gene using primers 515F and 806R and amplicon sequencing were performed on an Illumina MiSeq by the Biosciences Division Environmental Sample Preparation and Sequencing Facility (ESPSF) at Argonne National Laboratory (Lamont, IL).

### Sequence analysis.

Sequences were analyzed using QIIME (Quantitative Insights into Microbial Ecology) and R (R Project) (19, 20). Sequences were first demultiplexed and quality filtered using the default parameters, apart from a minimum Phred quality score of 25. Operational taxonomic units (OTUs) were chosen using uclust and the closed reference OTU picking method in QIIME, with 95% similarity (52). Taxonomic assignments were chosen by using PyNAST and aligning to the Greengenes database (13_8) (53, 54). Alpha diversity and UniFrac beta diversity, as well as the respective statistical analyses, were completed using QIIME (55). Taxonomic abundances were compared using a custom R script provided by the Institute for Genome Science, University of Maryland.

### PICRUSt analysis.

Predictive metagenomic functionality was determined by analyzing 16S rRNA gene sequences using PICRUSt (Phylogenetic Investigation of Communities by Reconstruction of Unobserved States) and STAMP (Statistical Analysis of Taxonomic and Functional Profiles) (28, 29). Because all but one of the piglets that showed evidence of disease were 6 days old or younger, piglets were grouped as younger (2 to 6 days) or older (8 to 14 days). Statistical analysis for two-group comparisons was completed using White’s nonparametric *t* test with Benjamini-Hochberg false discovery rate (FDR) multiple-test corrections.

### Data availability.

Data are publicly available in the NCBI SRA under accession number PRJNA730839.
